# Response of chia (*Salvia hispanica*) to sowing times and phosphorus rates over two crop cycles

**DOI:** 10.1016/j.heliyon.2020.e05051

**Published:** 2020-09-24

**Authors:** Tiago Roque Benetoli da Silva, Sheila Castro de Melo, Andressa Bezerra Nascimento, Lucas Ambrosano, Jaqueline Calzavara Bordin, Charline Zaratin Alves, Deonir Secco, Reginaldo Ferreira Santos, Affonso Celso Gonçalves-Jr, Gessica Daiane da Silva

**Affiliations:** a Universidade Estadual of Maringa – UEM, Umuarama, Paraná State, Brazil; b Universidade of Oeste of Parana – UNIOESTE, Cascavel, Paraná State, Brazil; c Universidade Federal of Mato Grosso of Sul – UFMD, Chapadão do Sul, Mato Grosso do Sul State, Brazil; d Universidade of Oeste of Parana – UNIOESTE, Marechal Cândido Rondon, Paraná State, Brazil

**Keywords:** Agricultural sciences, *Salvia hispanica*, Oilseed, Off-season, Phosphorus fertilization

## Abstract

Chia (*Salvia hispanica*) is an annual oilseed crop of high nutritional value. This study aimed to analyze the performance of chia plants sown at different times and grown under different phosphorus rates. The experiment was conducted under field conditions at the Experimental Farm of the State University of Maringá, Umuarama, Brazil. Treatments were arranged in a randomized split-plot design with three replications. Phosphorus fertilizer was applied at the time of sowing at rates of 0, 40, 80, or 120 kg P_2_O_5_ ha^−1^. Seeds were sown on four dates at 14-day intervals, starting on March 21, 2017, for the first crop and March 29, 2018, for the second crop. Plant population density, raceme number, shoot dry matter yield, thousand seed weight, seed yield, and seed oil content were determined. It was observed that the end of March is the borderline for chia cultivation, because the low temperature and rainfall conditions occurring after this period compromise chia growth. The best phosphorous rate for chia growth was 80–120 kg ha^−1^, which led to optimum vegetative parameters.

## 1 Introduction

Chia (*Salvia hispanica*) is an annual herbaceous plant belonging to the family Lamiaceae, which also includes mint, rosemary, oregano, and thyme (Coelho and Salas-Mellado, 2014; Garcez, 2013). Native to Mexico and Guatemala, chia is well adapted to tropical and subtropical climates (Capitani et al., 2012) but does not tolerate frost (Jiménez, 2010). Chia seeds were one of the main staple foods for pre-Columbian civilizations of Central America and currently find application in human and animal nutrition as well as in the pharmaceutical industry (Ayerza and Coates, 2004; Cahill, 2003).

Information on chia management is fairly limited (Migliavacca et al., 2014; Busilacchi et al., 2013), especially regarding soil conditions, sowing times, and fertilization. The plant is known to grow well in sandy, well-drained, slightly damp soils. It tolerates acidic soil, but growth is optimal at pH 6.5–8.5 (Pozo and Anabel, 2010).

Sowing time is a major factor influencing seed yield and other production parameters (Peixoto et al., 2000). Different species and cultivars can vary greatly in their response to environmental conditions and sowing times. Sowing time must be determined taking into account plant phenology and edaphoclimatic conditions.

In Paraguay, chia is cultivated both in the main season (December to January) and in the off-season (March to April) (Aguilera, 2013). In Argentina, it is typically sown in the first half of January (Busilacchi et al., 2013), whereas, in some regions of Brazil, it is sown in the off-season (Migliavacca et al., 2014).

Proper fertilization is economically and environmentally important, as minimizing nutrient loss to the environment reduces costs and increases productivity. Phosphorus is essential for energy transfer, maintenance of internal cell pressure, and enzyme activity (Souza and Chaves, 2017). In oilseeds, phosphorus fertilization contributes to increasing oil quality (Ramos et al., 2005). Maintaining adequate phosphorus levels is especially important at the early stages of plant development to avoid reduced growth, delayed leaf emergence, secondary root development, low dry matter production, and seed damage (Souza and Chaves, 2017; Grant et al., 2001).

Quantitative and qualitative analysis of plant development allows for a better understanding of plant responses to a given factor. This study aimed to evaluate the effect of sowing time and phosphorus fertilization on the production parameters of chia over two crop cycles.

## 2 Material and methods

### 2.1 Study site

The experiment was conducted under field conditions in 2017 and 2018 at the Experimental Farm (23°47′28.4″S, 53°15′24″W, 379 m above sea level) of the State University of Maringá, Umuarama, PR, Brazil. The soil is dystrophic sandy Oxisol (USDA, 1998). The local climate is classified as Cfa (humid subtropical), with an average temperature lower than 18 °C in the cooler months and higher than 22 °C in the warmer months. Frosts are rare, summers are hot and wet, and there is no marked dry season. Rainfall, temperature, and humidity data were collected from a local weather station and are presented in Table 1.

**Table 1:** Meteorological data of experiment conduction period in 2017 and 2018.

Month	Total preciptation (mm)	Minimum temperature (^o^C)	Maximum temperature (^o^C)	Relactive humidity (%) Average
**2017**				
March∗	15.6	18.9	30.2	71.2
April	134.3	19.8	31.1	73.5
May	247.7	18.0	25.2	82.9
June	69.1	14.8	24.2	72.9
July∗∗	1.0	12.1	24.8	54.2
**2018**				
March∗∗∗	214.5	19.0	35.0	56.2
April	32.9	17.3	33.0	60.1
May	61.4	8.3	32.5	58.5
June	30.5	8.0	30.2	75.1
July	8.0	5.3	32.6	54.6
August∗∗∗∗	160.8	6.6	33.7	66.1

Source: (SEAB, 2017 and 2018).

S1, S2, S3 and S4 = 03/21, 04/04, 04/18 and 05/02/2017, respectively.

S1, S2, S3 and S4 = 03/29, 04/12, 04/26, 05/10/2018 respectively.

∗ From day 03/21/17 (seedin).

∗∗ until 07/20/17 (harvest).

∗∗∗ From day 04/29/2018 (seeding).

∗∗∗∗ until 08/20/2018 (harvest).

### 2.2 Experiment

Ninety days before the beginning of the experiment, soil samples were collected at the 0–20 cm depth for analysis. Initial soil parameters are presented in Table 2.

**Table 2:** Soil chemical attributes of the experiment site, in the 0–20 cm layer.

pH	P	M.O.	Ca	K	Mg	Al	CCC	V
CaCl_2_	mg dm^−3^	g dm^−3^	cmol_c_ dm^−3^					%
4.35	1.0	15.04	0.72	0.1	0.21	1.35	3.88	26.55

Chia seeds were purchased from a local seed company. Prior to sowing, dolomitic limestone was applied to the soil to increase base saturation to 70% (Maia and Furlani, 1997). As there is no fertilizer recommendation for chia, we used the recommended rates for mint, which belongs to the same botanical family. Nitrogen fertilizer was applied at 20 and 30 kg ha^−1^ at the time of sowing and after mulching, respectively, and K_2_O was applied at 60 kg ha^−1^ at the time of sowing only (Maia and Furlani, 1997).

Treatments were arranged in a randomized split-plot design with three replications. Four sowing dates at 14-day intervals (main plots) and four rates (0, 40, 80, and 120 kg ha^−1^) of phosphate fertilizer (P_2_O_5_, subplots) were used per cycle, totaling 48 plots. Each experimental plot consisted of five rows, 5 m long and 0.45 m apart, planted with 25 seeds m^−1^ (3 kg ha^−1^). In the first crop cycle (2017), chia seeds were sown on March 21 (S_a_1), April 4 (S_a_2), April 18 (S_a_3), and May 2 (S_a_4). In the second crop cycle (2018), the sowing dates were March 29 (S_b_1), April 12 (S_b_2), April 26 (S_b_3), and May 10 (S_b_4). Phosphate fertilizer was broadcast before planting. Weeds were manually removed when necessary.

Plants were manually harvested from the center three rows of each subplot; outer rows were not sampled to avoid border effects. A late autumn frost was recorded on July 18, 2017, and all plots were harvested on July 20. The number of harvested plants was counted before harvesting in the useful area of each subplot. Population density was calculated for each subplot as the number of plants in center rows, according to Rogério et al. (2012). Inflorescences were counted to determine the number of racemes per plant. Seeds were threshed and cleaned by sieving. After threshing, shoots were dried in a forced-air oven at 65 °C for 48 h and weighed to calculate the shoot dry matter yield (t ha^−1^).

Seed moisture content was adjusted to 13% for determination of thousand seed weight and seed yield (Brasil, 2009). Thousand seed weight was determined by counting and weighing two replicates of 1000 seeds per plot. Seed yield (kg ha^−1^) was calculated from the weight of harvested seeds per plot.

Oil content was determined by Soxhlet extraction with hexane (IAL, 2008).

### 2.3 Statistical analysis

Data were subjected to analysis of variance at the 5% significance level. Season traits were compared by Tukey's test at the 5% significance level. Linear and quadratic regression models were developed to describe the relationship between phosphorus rates and production parameters. Statistical analyses were performed using SISVAR version 5.6 (Ferreira, 2011).

## 3 Results and discussion

### 3.1 Plant population density, raceme number, and shoot dry matter yield

Water supply was adequate throughout the first and second crop cycles (Table 1). Total rainfall was 467.7 mm in 2017 and 508.1 mm in 2018. Chia can grow under dry and wet conditions (300–1000 mm rainfall per year) but requires moist soil to germinate (Ayerza and Coates, 2005; Yeboah et al., 2014). The oilseed has optimal growth at 16–26 °C, tolerating minimum and maximum temperatures of 11 and 36 °C, respectively (Coates and Ayerza, 1996). The occurrence of frost on June 18, 2017, affected the development of plants grown in S_a_3 and S_a_4. Early flowering was induced, leading to variations in plant size and low raceme number, shoot dry matter yield, and thousand seed weight (Coates, 2011; Win et al., 2018).

In the first crop cycle, sowing time had significant effects on plant population density, raceme number, and shoot dry matter yield. The interaction effects of sowing time and phosphorus rate, however, were not significant (Table 3).

**Table 3:** Plants final population (ha), racemeper plant and shoot dry matter (t ha^−1^) of chia plants as function of sowing times. Umuarama (PR-Brazil) – 2017.

Treatments	Final population	Raceme/plant	Dry matter
	plants ha^−1^	number	t ha^−1^
**Sowing times (S)**			
S1	1,025,926 a	5.6 a	7.7 a
S2	805,555 ab	4.5 a	4.9 b
S3	924,074 ab	3.7 ab	5.1 b
S4	616,667 b	2.0 b	3.5 c
C.V. plot (%)	22.1	12.3	11.4
C.V. subplot (%)	33.1	27.1	19.6
**F test**			
S	∗∗	∗∗	∗∗
Rates (R)	n.s.	∗	∗
SxR	n.s.	n.s.	n.s.
L.R.	n.s.	n.s.	n.s.
Q.R.	n.s.	∗	∗

S1, S2, S3 and S4 = 03/21, 04/04, 04/18 and 05/02/2017, respectively.

Averages followed by the same letter in the column do not differ from each other by the Tukey test at 5% probability.

C.V. = Coeficiente of variationL.R. and Q.R. = Linear and quadratic regression, respectively.

∗, ∗∗ and n.s. = significant at 5, 1% and not significant, respectively.

Plant population density was higher in S_a_1 than in S_a_4 (Table 3). According to Melgarejo et al. (2014), uniform plant populations have higher seed yield potential. The low population density in S_a_4 was likely due to low temperatures, which delays chia seed germination and growth (Capitani et al., 2012; Sanchez et al., 2014).

Raceme number was lowest in S_a_4 and highest in S_a_1 and S_a_2, not differing between the two (Table 3). Plants grown in S_a_3 and S_a_4 did not show fully developed racemes and consequently had low seed yields. The results indicate that assimilate utilization was low, affecting raceme development (Baginsky et al., 2016). Karim et al. (2015) observed that chia plants sown in November had higher raceme numbers than plants sown in December, January, February, and March in Bangladesh. The authors attributed these results to favorable climatic conditions and higher plant height.

Chia is a short-day, tropical-climate plant. It grows between latitudes 21° and 25° and blooms only under a photoperiod of less than 12.5 h light (Sorondo, 2014; Jamboonsri et al., 2012). The study region (Umuarama, Brazil) is located at 23°47′S and has a subtropical climate. The photoperiod ranged from 11 to 12 h light, which probably contributed to plant development. In S_a_1, temperatures were more favorable for germination and growth.

The time from sowing to harvest was 123 days in S_a_1 and 80 days in S_a_4. Consequently, shoot dry matter yield was higher in S_a_1 (Table 3). Dry matter is an indication of crop productivity and is influenced by several factors, including sowing time. In the study of Karim et al. (2015), carried out in Mymensingh, Bangladesh (24°44′N), the highest dry matter yield was observed in plants sown in November. Differences in latitude, photoperiod, soil conditions, and fertilization must be taken into account for comparison of results.

In the second crop cycle, the interaction effects of sowing time and phosphorus rates were not significant (Table 4). Sowing time significantly influenced all parameters, except raceme number. Plant population was higher in S_b_1 and S_b_2. High plant population densities reduce evaporative water loss and competition with weeds, thereby increasing seed yield, soil cover, and nutrient uptake efficiency as a result of intraspecific competition (Tourino et al., 2002). Similar to the observed in the first crop cycle, shoot dry matter yield was lower in S_b_3 and S_b_4 because of exposure to low temperatures at early developmental phases (Table 4).

**Table 4:** Plants final plant population (ha), raceme per plant and shoot dry matter (t ha^−1^) of chia plants as function of sowing times. Umuarama (PR-Brazil) – 2018.

Treatments	Final population	Raceme/plant	Dry matter
	plants ha^−1^	number	t ha^−1^
**Sowing times (S)**			
S1	620,370 a	5.9 a	6.9 a
S2	598,148 a	5.2 a	3.0 b
S3	461,111 b	6.0 a	2.1 bc
S4	285,185 c	5.6 a	1.5 c
C.V. plot (%)	11.3	14.3	22.1
C.V. subplot (%)	17.4	27.7	30.3
**F test**			
S	∗∗	n.s.	∗∗
Rates (R)	n.s.	∗	∗∗
SxR	n.s.	n.s.	n.s.
L.R.	n.s.	n.s.	n.s.
Q.R.	n.s.	∗	∗∗

S1, S2, S3 and S4 = 03/29, 04/12, 04/26, 05/10/2018 respectively.

Averages followed by the same letter in the column do not differ from each other by the Tukey test at 5% probability.

C.V. = Coeficiente of variation.

L.R. and Q.R. = Linear and quadratic regression, respectively.

∗, ∗∗ and n.s. = significant at 5, 1% and not significant, respectively.

### 3.2 Thousand seed weight, seed yield, and seed oil content

In the first crop cycle, thousand seed weight did not differ between S_a_1 and S_a_2 and was lowest in S_a_4 (Table 5). This parameter is related to seed yield (Grimes et al., 2018). The climatic conditions were suitable for crop development, temperatures were above 10 °C, and rainfall was adequate (Table 1), similar to the conditions observed in a study by Grimes et al. (2018).

**Table 5:** 1,000 seed mass (g), yield (kg ha^−1^) oil content (%) on chia seeds, as function of sowing times. Umuarama (PR) – 2017.

Treatments	1.000 seed mass	Yield	Oil content
	(g)	(kg ha^−1^)	%
**Sowing times (S)**			
S1	11.2 a	1,115 a	7.3 a
S2	11.3 a	627 b	9.1 a
S3	10.9 ab	416 bc	5.6 a
S4	10.4 b	113 c	6.4 a
C.V. plot (%)	4.6	44.3	35.3
C.V. subplot (%)	2.3	33.1	35.8
**F test**			
S	∗	∗∗	n.s.
Rates (R)	n.s.	∗	∗
SxR	n.s.	n.s.	n.s.
L.R.	n.s.	n.s.	∗∗
Q.R.	n.s.	n.s.	n.s.

S1, S2, S3 and S4 = 03/21, 04/04, 04/18 and 05/02/2017, respectively.

Averages followed by the same letter in the column do not differ from each other by the Tukey test at 5% probability.

C.V. = Coeficiente of variation.

L.R. and Q.R. = Linear and quadratic regression, respectively.

∗, ∗∗ and n.s. = significant at 5, 1% and not significant, respectively.

Seed yield was highest in S_a_1. Plants had larger inflorescences, indicative of their production potential. The sowing date is highly relevant, as it determines the period of crop development and flowering induction. Variations in temperature and day length influence biomass production and seed quality (Grimes et al., 2018; Wojahn et al., 2018). As a short-day plant, chia has a critical photoperiod of 12–13 h light (Wojahn, 2016; Jamboonsri et al., 2012). The results indicate that the best growing conditions occurred during S_a_1.

Thousand seed weight varied significantly among sowing times in the second crop cycle; the highest values were observed in S_b_1 and S_b_2 (Table 6). The low temperature and humidity conditions observed in S_b_3 and S_b_4 probably affected seed development. The results corroborate the findings of Cahill and Ehdaie (2005), who reported that earlier sowing times lead to better production performance.

**Table 6:** 1,000 seed mass (g), yield (kg ha^−1^) oil content (%) on chia seeds, as function of sowing times. Umuarama (PR) – 2018.

Treatments	1.000 seed mass	Yield	Oil content
	(g)	(kg ha^−1^)	%
**Sowing times (S)**			
S1	8.3 a	987 a	6.4 a
S2	8.1 a	392 b	5.6 a
S3	7.9 ab	377 b	6.4 a
S4	7.7 b	334 b	5.9 a
C.V. plot (%)	3.3	28,3	17.7
C.V. subplot (%)	4.1	29,9	19.2
**F test**			
S	∗∗	∗∗	n.s.
Rates (R)	n.s.	∗∗	∗∗
SxR	n.s.	n.s.	n.s.
L.R.	n.s.	n.s.	∗∗
Q.R.	n.s.	∗∗	n.s.

S1, S2, S3 and S4 = 03/29, 04/12, 04/26, 05/10/2018 respectively.

Averages followed by the same letter in the column do not differ from each other by the Tukey test at 5% probability.

C.V. = Coeficient of variation.

L.R. and Q.R. = Linear and quadratic regression, respectively.

∗, ∗∗ and n.s. = significant at 5, 1% and not significant, respectively.

Chia had low performance in the current study compared with the crop's known potential. In a study by Grimes et al. (2018), the seed yield of different chia genotypes grown in Europe ranged from 100 to 1290 kg ha^−1^, showing that chia seed yield may vary greatly.

Sowing time did not influence the seed oil content of the first crop (Table 6), indicating that this variable was not affected by temperature and humidity conditions. In a study conducted in five different regions of northeastern Argentina, Ayerza (1995) found that chia seed oil content and composition varied with location. Higher seed oil content was observed in plants grown under favorable growth conditions, such as temperatures above 10 °C and adequate rainfall. In addition to climatic conditions, soil type and nutrition may influence the oil content of oilseeds (Ayerza, 1995).

Seed oil content was shown to be significantly influenced by sowing time in different oilseed crops. This was due to the scarcity of rainfall after April (Table 1), which influenced the final seed oil content. Melgarejo et al. (2014), in a study carried out in Marechal Cândido Rondon, Brazil, found that late sowing reduced the seed oil content of canola. Thomaz et al. (2012) reported similar findings. The authors planted sunflower seeds in July 2007 and January 2008 in Ponta Grossa, Brazil, and observed lower seed oil content in plants sown in the later season. We highlight that extraction method may also influence oil yield (Rodrigues, 2016).

The vegetative and reproductive development of chia was lower in the second crop compared with the first crop cycle because of differences in climatic conditions. Temperature and rainfall were lower during the flowering stage, which occurred from May to June (Table 1). Grimes et al. (2018) found that temperatures above 16 °C provide optimum conditions for plant performance.

### 3.3 Relationship between phosphorus rates and production parameters

**Figure 1:**
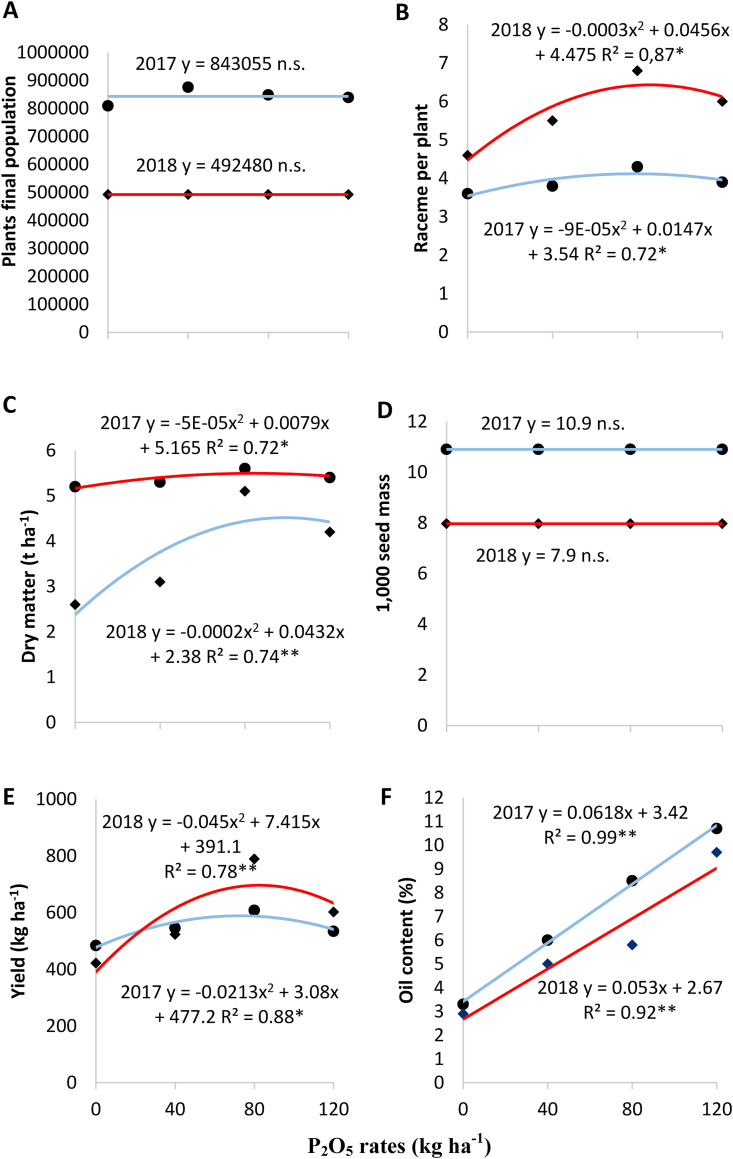
(A) Plants final population, (B) raceme per plant, (C) shoot dry matter (t ha^−1^), (D) 1,000 seed mass (g), (E) yield (kg ha^−1^) and (F) oil content (%) on chia plants as function of phosphorus rates. Umuarama (PR-Brazil) – 2017 and 2018. ∗, ∗∗ an n.s. = significant at 5, 1% and not significant, respectively.

In the first crop cycle, phosphorus rates did not influence plant population density (Figure 1a).

The relationship between raceme number and phosphorus rate was explained by a quadratic equation *y* = −0.0009*x*^2^ − 0.0147*x* + 3.4 (Figure 1b); the model showed that the highest raceme number is achieved using 81.7 kg P_2_O_5_ ha^−1^. Chan (2016) investigated the effect of phosphorus fertilization on chia growth and found that the highest raceme number was achieved using 90 kg ha^−1^. Phosphorus promotes photoassimilate production, as it favors leaf and raceme development (Taiz and Zeiger, 2013).

Souza and Chaves (2017) investigated the effects of nitrogen, phosphorus, and potassium rates on the development of chia grown under greenhouse conditions. The number of inflorescences per plant increased with increasing nutrient rates. The application of 125, 100, and 40 kg ha^−1^ of N, P_2_O_5_, and K_2_O, respectively, produced the best results.

Shoot dry matter yield had a quadratic relationship with phosphorus rate (*y* = −0.0005*x*^2^ – 0.0079*x* + 51.65), as shown in Figure 1c. A phosphorus rate of 79 kg ha^−1^ provides the highest dry matter yield, according to the regression model. Souza and Chaves (2017) found that higher shoot dry matter was obtained using 71 kg P_2_O_5_ ha^−1^ on plants harvested after 60 days, in agreement with the findings of the present study. Mint shoot growth was favored by fertilization with 24 and 30 mg L^−1^ phosphorus in a study by Rodrigues et al. (2004). Blank et al. (2006) found that lack of phosphorus fertilization decreased peppermint dry matter yield. According to Grant et al. (2001), phosphorus deficiency leads to reduced dry matter and seed production.

Phosphorus rates did not influence thousand seed weight (Figure 1d), possibly because phosphorus did not increase seed density; rather, it increased the number of seeds per plant, thereby increasing seed yield (Rogério et al., 2012). In sesame, thousand seed weight increased with phosphorus rates (0–120 kg P_2_O_5_ ha^−1^); the highest phosphorus rate led to a 14% increase in thousand seed weight compared to the control (Carneiro et al., 2016).

The second-order regression model for seed yield (*y* = −0.0213*x*^2^ + 3.08*x* + 477.2) showed that the maximum value is achieved using 72.3 kg P_2_O_5_ ha^−1^ (Figure 1e). This result contradicts literature data on other oilseed crops. Carneiro et al. (2016) found that 120 kg P_2_O_5_ ha^−1^ afforded the highest sesame seed yield. Sachs et al. (2006) evaluated the influence of NPK rates and found that sunflower seed yield increased by using 55 kg N ha^−1^, 41 kg K_2_O ha^−1^, and 46 kg P_2_O_5_ ha^−1^. Soares et al. (2016), on the other hand, reported that 100 kg P_2_O_5_ ha^−1^ increased sunflower seed yield by 58%.

Seed oil content (Figure 1f) increased linearly with phosphorus rates (*y* = 0.0618*x* + 3.42), which is probably due to the important role of this nutrient in metabolic pathways (David et al., 2006). Phosphorus is a constituent of DNA and RNA molecules, has an important role in protein synthesis (Taiz and Zeiger, 2013), and participates in the biosynthetic pathways of lipid formation from mono- and sesquiterpenes (David et al., 2007).

In the second crop cycle, there was no difference in plant population density between phosphorus rates (Figure 1a). However, as shown in Figure 1b, the number of racemes per plant was influenced by phosphorus fertilization. The relationship between raceme number and phosphorus rates were described by a quadratic equation (*y* = −0.0003*x*^2^ + 0.0456*x* + 4.475), revealing that the highest raceme number is achieved with 76 kg P_2_O_5_ ha^−1^. In a study carried out by Banjaña (2016) in Ecuador, chia plants fertilized with 60 kg P_2_O_5_ ha^−1^ had higher raceme number than those treated with 30 kg P_2_O_5_ ha^−1^. Chan (2016) reported that a phosphorus rate of 90 kg ha^−1^ is optimal for raceme production. Phosphorus has a key role in plant growth, development, leaf emergence, respiration, and photosynthesis (Taiz and Zeiger, 2013); therefore, its influence on raceme development is not surprising. Low phosphorus availability leads to reduced plant height and raceme production (Chan, 2016).

The variation in shoot dry matter yield as a function of phosphorus rates is presented in Figure 1c. A quadratic equation (*y* = −0.0002*x*^2^ + 0.0432*x* + 2.38) provided the best fit to the data and revealed that the optimal phosphorus rate for shoot dry matter yield is 108 kg ha^−1^ Souza et al. (2012), studying the effect of phosphorus fertilization on the biomass yield of Lamiaceae species, found that application of 180 kg P_2_O_5_ ha^−1^ increased root, shoot, and leaf dry weight. In an experiment with peppermint fertilized with phosphorus solutions (46.5, 31, and 15 mg L^−1^), David et al. (2007) observed that plant dry weight increases with increasing phosphorus rates. According to Novais and Smyth (1999), phosphorus fertilization is important in Brazil, where soils typically have low phosphorus availability.

As also observed in the first crop cycle, thousand seed weight was not influenced by phosphorus rates in the second cycle (Figure 1d). Seed yield, on the other hand, had a quadratic relationship with phosphorus rate (*y* = −0.045*x*_2_ + 7.415*x* + 391.1) (Figure 1e). According to the model, the maximum seed yield is achieved with 82 kg P_2_O_5_ ha^−1^. Zucareli et al. (2006) investigated the effect of six phosphorus rates (0, 30, 60, 90, 120, and 150 kg P_2_O_5_ ha^−1^) on bean development and reported that fertilization had no effect on thousand seed weight but seed yield and pod number were highest at 150 kg P_2_O_5_ ha^−1^. In soybean, seed yield was influenced by phosphorus fertilization (0–160 kg ha^−1^); the highest yield was achieved using 160 kg ha^−1^ (Batistella et al., 2013). Eltz et al. (2010) concluded that 80 kg ha^−1^ is sufficient to increase sunflower seed yield, whereas Soares et al. (2016) reported that the ideal rate is 100 kg ha^−1^.

Oil content (Figure 1f) increased linearly with phosphorus rates (*y* = 0.053*x* + 2.67). Phosphorus has a major role in energy metabolism and, consequently, in lipid synthesis, as it a component of ATP, ADP, and NADPH (Ramezani et al., 2009). A phosphorus rate of 120 kg P_2_O_5_ ha^−1^ increased the seed oil yield of chamomile (*Chamomilla recutita* L.) by 92% compared to the control. In contrast, phosphorus fertilization (24 and 30 mg L^−1^) reduced essential oil content in peppermint (Rodrigues et al., 2004).

In the present study, seed oil content was low, regardless of phosphorous fertilization rates, in comparison with seed oil contents (about 30%) reported by Grimes et al. (2018). However, the studied chia variety was new and was planted for the first time in Brazil. Thus, no prior information on its performance in the studied area was available. This is the first study to report the performance of chia in southern Brazil (Umuarama, Paraná, Brazil).

## 4 Conclusion

The findings of the current study indicate that the optimum sowing time for chia in southern Brazil is March, as planting after this month may influence plant growth because of nonoptimal photoperiod, temperature, and humidity conditions. The ideal range of phosphorus rates for good vegetative and reproductive development is 80–120 kg ha^−1^.

## Declarations

### Author contribution statement

T. R. B. da Silva: Conceived and designed the experiments; Analyzed and interpreted the data; Wrote the paper.

S. C. de Melo, A. B. Nascimento: Performed the experiments; Analyzed and interpreted the data; Wrote the paper.

L. Ambrosano: Conceived and designed the experiments; Analyzed and interpreted the data.

J. C. Bordin: Performed the experiments.

C. Z. Alves, D. Secco, R. F. Santos, G. D. da Silva: Analyzed and interpreted the data; Wrote the paper.

A. C. Gonçalves-Jr: Contributed reagents, materials, analysis tools or data; Wrote the paper.

### Funding statement

T. R. B. da Silva was supported by a research productivity scholarship from the Brazilian National Council for Scientific and Technological Development (CNPq).

### Competing interest statement

The authors declare no conflict of interest.

### Additional information

No additional information is available for this paper.
